# Opportunities and challenges in implementing community based skilled birth attendance strategy in Kenya

**DOI:** 10.1186/1471-2393-14-279

**Published:** 2014-08-15

**Authors:** Margaret Titty Mannah, Charlotte Warren, Shiphrah Kuria, Adetoro A Adegoke

**Affiliations:** Ministry of Health and Sanitation, Freetown, Sierra Leone West Africa; Population Council, Ralph Bunche Road, Nairobi, Kenya; Reproductive Health Division, Ministry of Public Health and Sanitation, Nairobi, Kenya; Liverpool School of Tropical Medicine, Pembroke Pl Liverpool, Merseyside, L3 5QA UK

**Keywords:** Community based skilled birth attendance, Skilled birth attendants, Community midwifery, Kenya, Maternal health, Safe motherhood, MDG5

## Abstract

**Background:**

Availability of skilled care at birth remains a major problem in most developing countries. In an effort to increase access to skilled birth attendance, the Kenyan government implemented the community midwifery programme in 2005. The aim of this programme was to increase women’s access to skilled care during pregnancy, childbirth and post-partum within their communities.

**Methods:**

Qualitative research involving in-depth interviews with 20 community midwives and six key informants. The key informants were funder, managers, coordinators and supervisors of the programme. Interviews were conducted between June to July, 2011 in two districts in Western and Central provinces of Kenya.

**Results:**

Findings showed major challenges and opportunities in implementing the community midwifery programme. Challenges of the programme were: socio-economic issues, unavailability of logistics, problems of transportation for referrals and insecurity. Participants also identified the advantages of having midwives in the community which were provision of individualised care; living in the same community with clients which made community midwives easily accessible; and flexible payment options.

**Conclusions:**

Although the community midwifery model is a culturally acceptable method to increase skilled birth attendance in Kenya, the use of skilled birth attendance however remains disproportionately lower among poor mothers. Despite several governmental efforts to increase access and coverage of delivery services to the poor, it is clear that the poor may still not access skilled care even with skilled birth attendants residing in the community due to several socio-economic barriers.

**Electronic supplementary material:**

The online version of this article (doi:10.1186/1471-2393-14-279) contains supplementary material, which is available to authorized users.

## Background

One of the biggest health risks to women is pregnancy and childbirth. Each year, 358,000 women die due to preventable maternal causes [1]. Over 90% of maternal deaths occur in sub-Saharan Africa due to obstetric complications that could be managed effectively by increasing women’s access to Skilled Birth Attendance (SBA) [2].

Approximately 2 million babies also die each year due to complications during birth and the burden is inequitably carried by the poor [2]. Evidence-based strategies are therefore urgently needed to reduce the burden of intrapartum-related deaths especially in low income settings, where 60 million women mostly give birth at home every year [3].

Furthermore, it is suggested that 74% of maternal deaths and 63% of newborn deaths and disabilities could be averted if all births are attended by a Skilled Birth Attendant with access to a quality referral facility [4]. SBA however remains a major problem in developing countries, as 34% of deliveries globally still take place without a Skilled Birth Attendant, which translates into 45 million births [5]. Such a discrepancy poses a huge challenge to meeting the maternal health Millennium Development Goal (MDG 5) by 2015 [6].

SBA has been defined as the process where a skilled provider, supported by an enabling environment is able to support women with adequate care during labour, delivery and early postpartum period [6]. SBA has two components which are trained personnel (skilled birth attendant); and the enabling environment which includes availability of drugs, supplies, equipment, infrastructure and the right policies [6, 7].

Kenya has a high maternal mortality ratio of 488 per 100,000 live births [8] and a life time risk of maternal deaths of 1 in 38 [1]. Most women in Kenya deliver at home and only 44% of deliveries are attended by skilled attendants [8]. It is acknowledged that there is low utilisation of skilled providers during pregnancy, childbirth and the postnatal periods and limited provision of basic Emergency Obstetric and Newbon Care (EMoNC) exist [9].

The situation in Kenya is compounded by critical shortage of human resources for health. For example there are about 5,000 doctors in Kenya, for a population of 40 million, i.e. about 6,400 inhabitants per doctor [10]. There are 30,212 registered nurse-midwives and 4,813 enrolled nurse-midwives which are equivalent to 101 registered nurses-midwives and 16 enrolled nurses-midwives per 100,000 populations [11].

In a bid to address these gaps, the government of Kenya developed the community midwifery programme in 2005 as an additional strategy to increase access to skilled attendance at birth [Warren and Runbold: A review of community midwifery model in Western province, Kenya, unpublished report]. This programme was seen as an intervention targeting the more vulnerable, poor and rural populations. The aim of the programme was to link skilled birth attendants living within the community near women to facilitate skilled care during childbirth, and to refer complications to EmONC facilities nearby [Warren and Runbold: A review of community midwifery model in Western province, Kenya, unpublished report].

Retired and unemployed nurse-midwives were recruited and trained in focused antenatal care; management of normal labour and the use of the Partogram; EmONC and Life Saving Skills (LSS); postnatal care for mothers; essential newborn care; family planning; referral; infection prevention; interpersonal communication; and monitoring and record keeping. They were commissioned to provide care within the community and were attached to health facilities where they could refer their clients to when complications occur. Following successful completion of these refresher trainings, each community midwife (CM) was provided with a birth kit to assist births in the client’s home or in the midwife’s home in a culturally appropriate manner.

CMs were expected to work with their community to determine how they will be remunerated and it was generally agreed by CMs and the communities that clients can either pay CMs in cash (either in full or by instalment) or in kind (either by giving clothes, soaps) or working on the farm of the CMs [Warren and Runbold: A review of community midwifery model in Western province, Kenya, unpublished report].

In this study, we evaluate the Kenya community midwifery programme by exploring CMs and key informants’ experiences of the programme to determine its contribution in improving access to skilled care; and to identify opportunities and challenges that affect women’s access to skilled care. Although we assessed clients’ satisfaction with the care and services provided by CMs, findings of this will be presented in another paper.

## Methods

This study was conducted between June and July 2011. We used a qualitative approach utilising in-depth interviews (IDI) and key informant interviews (KII) to explore and understand different actors’ perspectives of the community midwifery programme.

Two topic guides were developed; these were the KII guide and IDI guide. The KII guide contained 12 major items which aimed to explore the views and experiences of key informants on the community midwifery programme and factors that enable or hinder the community midwifery programme (Additional file 1). The IDI guide on the other hand contained 18 key items exploring CMs experiences; performance; job satisfaction; opportunities; and challenges faced (Additional file 2). The topic guides were pre-tested for cultural relevance and appropriateness in the Webuye community in eastern part of Bungoma District, in Western province which was not included in the study (Topic guides are attached as additional files).

Triangulation of data was done by using these two data collection techniques to increase the validity of the study findings [12]. All interviews were conducted in English and were recorded using a digital audio recorder to ensure that all discussions were captured. MTM facilitated each interview and to improve the depth of the data collected, she was assisted by a Kiswahili/English research assistant, who was acquainted with the norms and culture of the area. Venues for interviews included participants’ homes, fields, clinics and church halls. The interviews lasted around 45 minutes to ensure prolonged engagement with participants. Summaries of interviews were made at the end of each interview to give opportunity to participants to agree with contents of their statements [13]. Notes were taken during field visits and interviews. The notes were expanded upon immediately afterwards.

### Study participants

The study participants included 20 CMs and six key informants. Key informants were selected from national, provincial and district levels of the Ministry of Public Health and Sanitation, and the Population Council. We used purposive sampling to recruit all key informants who were perceived to have knowledge about the community midwifery programme so as to generate useful data to respond to the key research questions [12]. Key informants for this study were the pioneers, funder, manager, coordinators and supervisors of the community midwifery programme. These include the programme coordinator based with the Population Council (funder), the programme manager within the Reproductive Health Division of the Ministry of Public Health, the two coordinators and two supervisors from both districts.

The CMs were selected from the registers maintained by the district supervisors. We ensured the inclusion of all CMs irrespective of location (either hard to reach or easily accessible areas) within the districts. As at the time of data collection, each district had 10 functional CMs and all were included in the study.

### Study site

The study sites were Kakamega District in the Western province and Maragua District in the Central province of Kenya. Of the two districts, Kakamega is under developed, with few public health facilities and skilled workers compared to Maragua.

The reproductive health status of women was extremely poor in the Western province as illustrated by low utilisation of SBA of 25.3%, high fertility rate of 5.6 which was above the national average. There was also a high incidence of teenage pregnancy of 15% compared with other regions [8]. In the Central province, although 73.8% of the women gave birth with the help of a skilled birth attendant [8, 14], there were areas within the province that had limited access to services. Contraceptive Prevalence Rate was high among married women at 67%, compared to a national average of 46% [8, 15] (Table 1).

**Table 1:** **Key characteristics of the study districts**

Indicator	Kakamega	Maragua
Proportion of births attended by Skilled Birth Attendants	25.8%	73.8%
Births delivered at health facility	25.3%	73%
Antenatal care attendance	91.5%	92.7%
Post natal care	40%	55.8%
Contraceptive prevalence rate	47%	67%
Fertility rate	5.6	3.4
Full immunisation coverage	73.1%	85.8%
Under 5 mortality	121 per 1000 live births	51 per 1000 live births

Reproductive, Maternal and Child Health Indicators for Kakamega District and Maragua District, Kenya.

During the study period, four non-governmental organisations were working in the two districts to support the government of Kenya in the field of maternal health. They were: The USAID funded AIDS Population Health Integrated Assistance II (APHIA II) programme; the Tunza project of Population Services International (PSI) on family planning; Essential Health Services and the Population Council.

### Ethical approval

Ethical approval for this study was granted by the Liverpool School of Tropical Medicine Research and Ethics Committee and the Scientific and Ethical Review Committee of the Kenyatta National Hospital in Kenya. Permission for the study was also obtained from the Division of Reproductive Health and the Director of each District Health Management Team.

Informed consent was obtained from all participants. They were informed about their right not to participate and to withdraw at any time. To maintain privacy, anonymity and confidentiality, names of places were coded and no names of participants were obtained [12].

### Data management and analysis

All audio recordings were transcribed. Each typed transcript was checked against the audio tape. All transcripts were later crossed checked with the recordings by the research team. Data were analysed using a thematic framework [13, 14]. Issues related to study aims were identified and coded without predefined categories capturing the main themes and concepts.

The coding process involved identifying major themes in each of the transcript. During data analysis, identified themes were compared across the transcripts and field notes to determine differences and similarities in the perspectives of the study participants. After coding, themes were developed and classified guided by a framework and a triangulation of data sources and methods. The process of triangulation was used to validate the findings. This involved comparing the identified themes from the IDIs and key informant interview transcripts [12]. Results were then written up thematically, organised around the main research questions.

### Qualitative research review guidelines (RATs)

This qualitative study has adhered to the guidelines for Qualitative research review guidelines (RATs).

## Results

All the participants willingly took part in the study with no one declining. The qualitative methodology promoted a 100% response rate and it gave opportunity for clarification and further probing.

Emerging themes from this study represent views and perceptions of the CMs and key stakeholders on their experiences with the community midwifery programme and the factors that enhance or inhibit access to community based skilled birth attendance. We have not disaggregated findings by location but have reported them as aggregates mainly by type of participants to avoid exposing participant’s identities. The result are organised into two dimensions with a range of issues emerging as opportunities and challenges of the community midwifery programme.

### Who are the CMs

Three of the 20 CMs and one of the six key informants were males. All CMs were registered nurse-midwives. Sixteen (80%) of the CMs were retired midwives and four (20%) were self-employed midwives before their recruitment into the community midwifery programme. The ages of CMs ranged between 40–60 years, with only five (25%) CMs between ages 40–45 years and the remaining 15 (75%) CMs were above 45 years. They all joined the community midwifery programme at its inception in 2005.

Findings of data from both CMs and key informants revealed that the community midwifery programme does not only involve retired midwives but also young and unemployed or self-employed midwives. All key informants stated that they were happy that their communities were fortunate to have skilled birth attendants whom they have worked with before retiring, with a lot of clinical experience and are willing to work for their communities. Key informants further explained that having young graduates was better than working with Traditional Birth Attendants (TBAs) as CMs knew exactly what to do and when to refer complications.

*“It is not just the old and the retired midwives that were recruited. We also found young ones (midwives) who were qualified from colleges but have not yet found a job. So we decided to include private and other midwives who are unemployed by the Government of Kenya to widen the pool of these midwives, which was good” KII_002*

*“We are finding it much easier to work with the CMs than what we were doing with the TBAs. It’s very interesting because they (CMs) are all qualified and are members whom we have worked with and we have seen them retire, we speak one language and we are able to understand one another” KII_003*

### Provision of individualised care

All participants stated that CMs offer antepartum, intrapartum and postpartum services, family planning counselling and services, HIV testing, health education and management of minor ailments.

Key informants also stated that services and care provided by CMs were individualised. In addition, they stated that CMs have very good relationship with the public. This was confirmed during the interviews with CMs, where-in counselling and health talks were given on a one to one basis. They stated that they spent long hours with their clients to have in-depth discussion of the care provided. Key informants and CMs stated that services were provided in a very humane manner and in a comfortable environment with no rush to complete within working hours.

*“We are readily available in the community. The community knows us well and we can easily reach the women in the community and educate them to access services more easily. We visit them at home, so you see” IDI_001*

### CMs increase women’s access to SBA in the community

All participants expressed that the community midwifery programme has increased access to skilled care especially for the poor and marginalised people in rural areas. All participants felt that the programme was very relevant and needs to be sustained and scaled-up in other areas of the country.

*“We are always there and available to them at any time…. It is even easier at night but only that we need an escort” IDI_005*

*“This programme has helped in getting services closer to women in their communities. It is very good of them to be within their localities.” KII_001*

### Opportunities and challenges to the community midwifery programme

**Figure 1:**
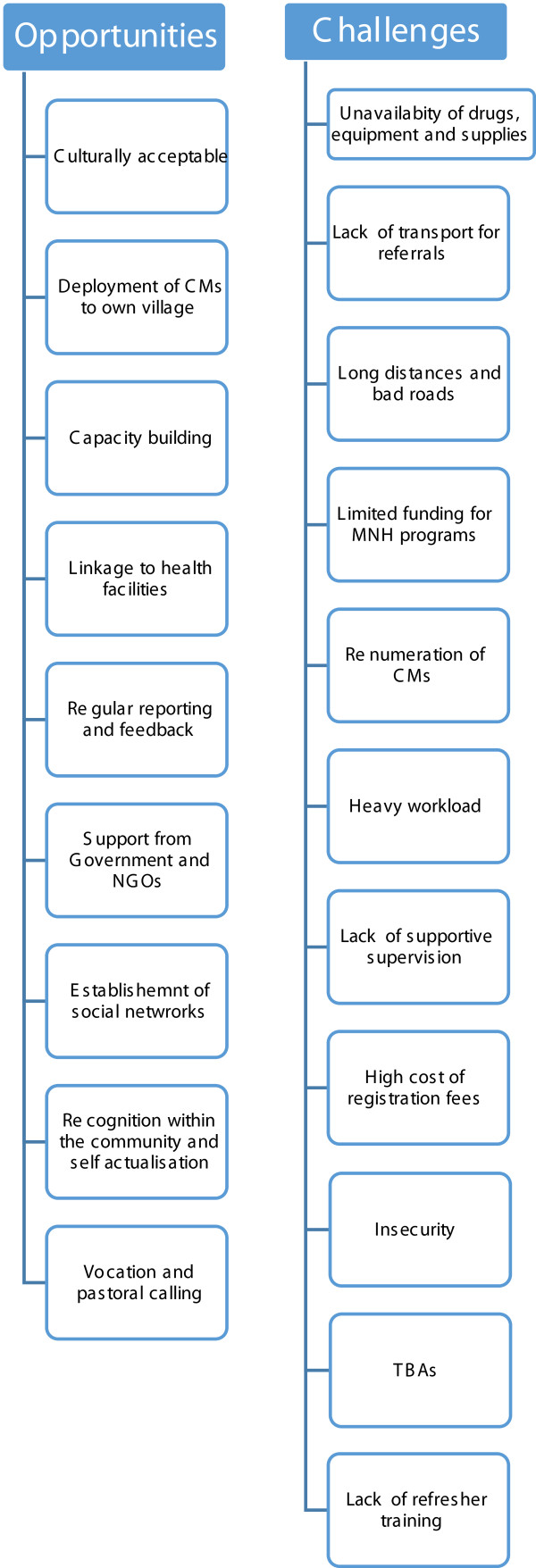
**Describes opportunities and challenges for the Kenya community based skilled birth attendance programme.** There were 9 themes identified as opportunities while, 11 themes were identified as challenges.

Participants described various challenges and opportunities to the community midwifery programme. These were categorised into themes. A total of nine themes were identified as opportunities while 11 themes were identified as challenges. Challenges to the community midwifery programme include: unavailability of drugs, equipment and supplies, lack of transport for referrals, long distances and bad roads, limited funding, inadequate remuneration, lack of supportive supervision, heavy workload, high cost of registration fees, insecurity, TBAs and lack of refresher trainings. Opportunities for the community midwifery programme were: a culturally acceptable intervention, deployment of CM to their own community, capacity building, linkage to health facilities, regular reporting and feedback, support from governmental and non-governmental organisations, establishment of social networks, recognition within the community, self-actualisation, vocational and pastoral calling (Figure 1).

### Opportunities

#### Culturally acceptable intervention

CMs assist women during childbirth, mainly in clients’ homes. All respondents indicated that the community midwifery programme offered an environment more conducive to childbirth and effective SBA in the community. This was stated by participants to be due to over 90% of the CMs being older women who command respect within their communities. In addition, CMs assist births in the clients own homestead rather than in CMs’ homes which allowed relatives to be around to give emotional and social support during labour. Assisting births in clients’ homes also allowed women and their relatives to dispose the placenta in a culturally acceptable manner. As an example, all the CMs in Western province and five of the CMs from central province described the culture of the Luya clan from Western province which only supports births at home especially for first births and places a lot of significance on where and how placentas are disposed. These were highlighted to be major cultural barriers to facility birth especially in Western province.

*“Like CMs at times you can be called in the home. So you carry a kit and assess the woman and if you see there is no complication you conduct the delivery at home. You work and their relatives are around to support them” IDI_005*

*“Some feel delivering in the village offers them privacy in a way in this society. As most of the CMs are experienced and age able (aged) women. So they like somebody like this going to deliver them in their homes. Yah” KII_006*

#### Deployment in own community

Key informants stated that CMs are the people best suited to work at community level because they understand their local customs. They further went on to say that one of the criteria for recruitment was that CMs must be residing in that community, this was confirmed by CMs responses.

CMs stated that they gave their contact details to clients and wrote their cell phone numbers on their doors and on clients’ cards. It was noted that this has made CMs more accessible to their clients and has reduced the cost of paying for a “matatu” or “boda boda” (minibus or motorbike/bicycle).

*“I reside from within my area and the community knows me very well. I write my phone number on their cards. Because I also stay within the community and my number is written on the clinic door, many of them have the number and they can call me anytime.” IDI_008*

#### Capacity building of CMs

Participants stated that Population Council helped with the initial training of CMs in EmONC and entrepreneur skills at the inception of the Community Midwifery programme. CMs also explained that the USAID funded APHIA II programme also helped with some refresher trainings of CMs in the Western province. CMs were happy that these trainings have increased their knowledge and skills in managing obstetric and newborn complications. CMs also noted that the initial training made them very enthusiastic and confident in performing their jobs. The entrepreneurial skills acquired were also reported to have helped CMs to be self-reliant and autonomous.

*“With support from XXXX, we then embarked on retraining and updating these CMs so that some who had lost skills are encouraged and retrained to conduct deliveries again. Now HIV, APHIA II is helping with training, so I feel it is very relevant because deliveries by SBA are increasing gradually and they are happy” KII_003*

#### Linkage with health facilities

All key informants stated that CMs were linked to health facilities which were to give support and supply CMs with some basic commodities and consumables. These facilities were also to help CMs with autoclaving of equipment and instruments. CMs stated that contact numbers of key health personnel and ambulance drivers were given to them for easy access during referrals. In addition, the health facilities involve CMs in some district health activities.

*“We link them up with the health facility nearby so that they will be able to assist them with commodities, equipment, supplies and also with where to autoclave or sterilize their equipment after they have used them” KII_002*

#### Regular reporting and feedback

Key informants stated that CMs work in collaboration with their linked government health facilities and send a monthly report of their activities to the district health team. CMs stated that they get feedback from the health facilities about referrals made. This helped in the provision of continuum of care on discharge.

*“We work as a group (team) with the Government of Kenya health facilities. They have to know what we are doing here by reporting. We give our District Public Health Nurse (DPHN) statistics of what we are doing every month. When we refer somebody, we give them a certain referral form. And then they also sometimes give us feedback to help us know when the woman comes back to the community, or if this and that has been done…” IDI_008*

#### Support from governmental and non-governmental organisations (NGOs)

All CMs and key informants stated that they were supported by NGOs with initial supply of delivery kits, supplies and family planning commodities. Essential Health Services and “TUNZA” project of Population Services International (PSI) also helped with the provision of family planning commodities as well as autoclave. These were given to CMs at subsidised or discount prices.

*“We were supported by a number of partners like Population Council and we also have the local partners like the APHIA II and TUNZA. A few of the CMs have been provided with delivery kits, stethoscopes, blood pressure machines and so on. We support them with immunization, we give them FP commodities for free and general supplies like gloves….” KII_003*

#### Establishment of social network (cluster) and regular monthly meetings

Key informants stated that CMs were encouraged to form social networks so that they would provide support to each other. The establishment of a social network helped them meet every month to discuss their welfare. During these meetings they also visited each other’s facility. CMs explained that the networks and regular meetings have helped them to get some equipment and to act as peer reviewers and auditors of the services and care rendered to clients.

*“Yes, we usually meet here every month with all CMs. we share our views, we say our problems and you are helped by the others. So we have been contributing some few shillings, coins like 700 shillings and then we had to buy each an autoclave. Also we usually go for visits. The TUNZA members also call us to air our views. If you have any problem you are helped, the marketing training is also helping, Eeem” IDI_009*

#### Recognition within the community and self-actualisation

CMs explained that the community midwifery programme has made them to be more recognised within their community. They stated that they felt satisfied and fulfilled when officials visited them from governmental and non-governmental organisations. CMs further explained that clients also showed appreciation giving maize, sugar and even working for them at home or in their farms. Some clients also paid the fees they were charged by CMs, although some did not pay. They explained that when they are going around in the community, people were happy to see them and some clients offered prayers for them.

*“I feel recognised somehow because before they would not even recognize me as a midwife, in the community. They only knew me as a nurse but not as a midwife. So I feel that I am recognized being a community midwife, I am happy” IDI_010*

*“You will find someone greeting me very warmly that “Sister, you really helped me. Thank you very much and may God bless you.” They shake my hand so hard until you find that my hand is very tired…..it’s an appreciation” IDI_004*

#### Vocation and pastoral work

CMs perceived the community midwifery programme as a mission, a calling from God. They explained that they were reaching out and serving humanity, which they found very motivating. CMs also explained that their commitment foster a sense of trust from women, made women to value their services, and encouraged women to return for additional visits.

*“I see myself as a pastor and I am called, so when I do my work, I don’t deserve any payments but after doing it, I just find someone bringing me something and I feel happy” IDI_007*

*“Most of them say that we are praying for you. They will just say thank you sister, I am grateful. God will help you. I think that’s why God is taking care of me so much” IDI_004*

### Challenges

Despite the opportunities and the positive effect of the community midwifery programme mentioned above, a broad set of challenges were revealed by all respondents in this study and these are described below:

#### Unavailability of drugs, basic equipment and supplies

CMs stated that they have not been receiving any supply of drugs and medical supplies. They stated that since their initial supply of delivery kits no one has re-issued a new one. Only the supplies of family planning commodities were continuously received from TUNZA project at a subsidised cost. They explained that lifesaving drugs such as Magnesium Sulphate for treatment of severe pre-eclampsia and eclampsia is not authorised at the community level. CMs stated that they do not have proper storage facilities for vaccines and Oxytocic drugs. As a result, clients had to be given prescriptions to buy drugs from local chemist shops including prescriptions for iron and vitamin supplements.

*“Some of the factors that I have encountered are problems with kits, delivery kits. Let’s say like myself I just have one, so I have to boil it and maybe I don’t have any one for emergency” IDI_009*

#### Lack of transport for referral

All respondents reported that it was very difficult to get transport to certain communities. They also explained that people mostly walk or use donkeys. During emergencies, CMs explained that most of the time they use stretchers or wheelbarrows to transport women with obstetric complications to health facilities. They further went on to say that hiring a “matatu” (public bus) is very expensive, inappropriate and sometimes impossible because some roads were impassable especially during the rains. CMs explained that when they call for an ambulance from the hospital, they were told that the ambulance was without fuel or that the patients should provide the fuel which most often was provided by the CMs to avoid further delays.

*“The biggest issues are the long distance....the means of transportation is poor…. Some people are forced to use a wheelbarrow to reach the road and hire a matatu which is very expensive” KII_002*

*“I once had a woman with complication, I suffered a lot. They are poor, there is no money, you have no vehicle, the place is so deep in the village, I really suffered. I called the ambulance and I told him (ambulance driver) that the woman is going to die in my clinic, please assist….he said I have no fuel. So I took my one thousand shillings and gave them” IDI_001*

#### Long distances and bad roads

CMs stated that even though they live in the community, they covered long distances on foot to reach some clients. They found this very challenging, especially when roads are bad. Some roads were also said to be impassable even with the use of motorbikes. This results most times in CMs incurring huge costs.

*“It is a big challenge because a mother in the house and she is 6 or more kilometres away and she calls you at night. Sometimes you are told to walk…my community is very hilly we have steep valleys and hills. Even bikes cannot pass easily, so it’s costly here. The long distances, bad roads and then you have been working the whole day; burn out actually, it’s a challenge. You are weary. The fatigue, the distances involved is a real challenge” IDI_001*

#### Limited funding for the community midwifery programme

All key informants stated that inadequate funds for maternal and newborn health programme have made some strategic health plans to be delayed or implemented ineffectively. They expressed concerns over the quality of services provided by CMs due to the fact that the government or NGOs have not been able to regularly replenish CMs initial birth kits and medical supplies. Some of the CMs stated that they feel guilty because they are unable to provide comprehensive antenatal services to clients. Some explained that they do not have refrigerators to store vaccines and certain life-saving drugs.

*“I do antenatal clinics but I feel inside me that I am not doing it completely because the other things like de-worming, prevention of malaria. I don’t do it because I don’t have them. Even for vaccines and oxytocin I send them to Government of Kenya facility, I don’t have a fridge, you see” IDI_001*

*“You see the country needs external funds to implement plans because we are having so many problems to solve with inadequate government funds…..which is also sometimes late” KII_003*

#### Remuneration

Key informants raised a concern that CMs have been expressing regarding their remuneration. They stated that volunteerism is at a cost especially when the person is not receiving support in terms of regular drugs and supplies. CMs complained about the financial burden of their work and the long hours with no schedule. In view of this, they expected some form of remuneration from the government. If the government is unable to provide remuneration, the CMs explained that they should be paid by the families of the clients or that the government should at least ensure regular replenishment of their birthing kits and supplies. CMs went further to explain that remuneration, when given at all, was always given with a great deal of reluctance by most clients.

*“Initially the services of the CM were meant to be voluntary services but then again it was not easy for the midwife to continue volunteering, so they charge some fee. You see, their children need food on the table and if they have to do that as a full time job, how will they feed their children? Volunteerism is too difficult, so that too has been a barrier” KII_003*

*“It has been difficult to work. You keep having the heart to work and to assist the community but you also need to be supported …..now some of our colleagues have left this job. You buy everything, with no salary they expect you to give services free of charge, how will I get “Unga” (flour) on my table…..” IDI_003*

#### Heavy work load

All the CMs in Maragua and four of the CMs in Kakamega explained that they have had to set up mini-clinics within their homes which they used when clients come to them. CMs were however the only one working in these mini clinics. CMs stated that they work both day and night attending to clients either in clients’ homes or in CMs’ homes. CMs stated that this heavy workload is tiring, and that it hinders them from concentrating on their farms or other income generating activities. Key informants and some CMs explained that some CMs have abandoned their job as a result of this workload and are engaged in other activities like farming.

*“Because they were coming and I could not turn them away. Someone will come and tells you that the wife’s placenta has stuck, how will you not go? You just have to go and assist that person. So whether I want to or not, I have to do it. This is making me not to do my farming work, you see. When I am here, I am just on duty day and night and always on call” IDI_009*

#### Lack of supportive supervision for CMs

Supervision of CMs was stated by key informants to be irregular and ineffective as a result of inadequate logistics. CM reiterated that they were not supervised regularly and that anytime district health team members visited them it was mostly for collection of returns and inspection.

*“The supervision is where we have not been doing very well because when you go to see them they expect something and you are not taking anything along, you see” KII_005*

#### High cost of registration fees to regulatory bodies

To practice as a midwife, CMs had to register with the regulatory body. All CMs on the community midwifery programme therefore had been registered to practice by the Nursing and Midwifery Council. Apart from this, CMs with clinics must obtain licence to open clinics from the Council. Failure to do so will lead to closure of clinic or withholding of licensure. This was confirmed from interviews with key informants.

*“They are also required to register with the regulatory body. The registration fee with the nursing council is about 10,000 Ksh per year.....So that has been a barrier” KII_003*

#### Insecurity

Insecurity was stated by all respondents as a major barrier. All participants explained that initially, the community agreed to provide CMs with escorts but this did not work well in some of the localities. In some localities, CMs stated that escorts were not provided for them at night, which was inhibiting their work as they were afraid of being attacked by thugs or being raped. This has made them not to want to attend to clients in their (clients) own homes at night.

*“We are called when it is already late at night. Insecurity is a very big problem and the escort that was to be allocated for me, I have never seen him. Now you would really want to go to attend them (to women) but you will look for escort but nowhere to get one” IDI_002*

*“I am willing to work for them (clients) always unless during the night because of thugs; when it’s already late at night; the insecurity is a very big problem. It’s not safe… ……. because you can even be raped you know” IDI_006*

#### TBAs

Key informants stated that one of the key reasons why the community midwifery programme was initiated to meet the needs of majority of women who want to deliver at home. CMs reported that even though there is now community based skilled birth attendant, some women still deliver at home with TBAs. CMs explained that TBAs only call for help when they encounter complications and most times these calls for help were very late.

*“So the bad is these TBA who confuse my clients by telling them not to come here because I am taking their work. These TBAs ask them (women) why they should go to the clinic to pay a lot of money when they are there. But at least they too are charging them. They stay with a client for a very long time and they bring to me when it’s very late.…..” IDI_008*

#### Lack of refresher training

CMs explained that since their initial training at the inception of the programme, they had not attended any further refresher trainings. Key informants confirmed this by expressing similar sentiments and were critical about the fact that CMs may not be current in their practice.

*“Well, I need training because since our first training in 2005, nobody has trained me again. I want to continue to be updated even though I am old you know (laughter)” IDI_007*

## Discussion

It has been evident that most obstetric complications occur around the time of delivery and cannot be predicted. In view of this, it is important that all pregnant women have access to a skilled attendant who is able to manage normal delivery and who can recognize and manage obstetric complications, or refer on time if needed [16–18].

The findings of this study affirm the value of providing skilled community based care at births. CMs are highly skilled and experienced midwives who provided maternal, newborn and child health care within their community. These retired or out of work CMs are able to provide competent, skilled and safe care closer to women and thus have the potential of reducing Kenya’s maternal and child mortality figures [19–22]. Community based skilled birth strategy has been described as a culturally acceptable method of supporting women during childbirth especially in areas where the proportion of facility birth is low and home delivery is high [7, 15].

Continuous professional education is critical to reinforcing knowledge and skills, and ensuring reduction in maternal and newborn mortality [23, 24]. Although training and capacity building are key elements to improving access to sustainable, high-quality maternal health services, most CMs have never had the opportunity to update their knowledge and skills after their initial training [25, 26]. The lack of training will have impact on the quality of care rendered and can result in possible demotivation for CMs. Continuous education of CMs is therefore critical and should be urgently prioritised.

Evidence has shown that lack of basic equipment and life-saving drugs are common problems in developing countries [27]. The unavailability of basic equipment, medical supplies and live saving drugs such as Magnesium Sulphate due to knowledge deficit and bureaucracies in health systems contributed to the demise of women in Kenya. This holds true in this study where CMs complained of unavailability of basic drugs, equipment and medical supplies. Life-saving, emergency drugs such as Magnesium Sulphate were not accessible to CMs partly because they were not trained to administer it or due to lack of policy supporting CMs to administer them at community level.

Wages and monetary benefits are important elements of recruitment and retention efforts of skilled personnel [4]. In addition, insufficient remuneration and unattractive working conditions are seen as major reasons for lack of access to health workers in rural areas [28]. CMs opting out of the programme and engaging in other activities not related to midwifery as a means of getting income has led to some communities not having CMs and clients having to walk long distances to access care. This is similar to findings from Tanzania, Bangladesh and another study in Kenya where inadequate remuneration and supplies discouraged Community Health Workers (CHWs) [29–32].

Health workers in the community have been referred to as the essential link in service delivery between primary health facilities and the communities [29]. This system has also emerged as a global attempt to revise primary health-care delivery in low-income settings and has been embraced by many resource limited countries such as Ethiopia, Tanzania, Pakistan, India, Brazil and Bangladesh as the cornerstone for community health delivery [33–35].

There are CHWs in Kenya, CMs however are not categorised as CHWs. CMs in Kenya are highly trained midwives working in the community and do not fit into the definition of CHWs. According to the WHO, CHWs are members of the community, who have been selected by, and are answerable to the communities where they work; supported by the health system; with less training than formally trained health workers [36].

In Kenya and many other low resource countries, CHWs have mostly been regarded as unpaid or poorly paid and lightly trained community members who volunteer to provide basic treatment, health education and promotion for their community. This unfortunate situation must have also influenced the lack of formal remuneration of CMs. In order to resolve CMs lack of income and reduce their attrition, the Kenyan government needs a review of its existing policies to come up with realistic strategies for staff retention in rural communities. The Kenya community strategy presents a good opportunity that can be explored further with the issue of remuneration. This strategy states that Community Health extension Workers (CHEW) and Community Health Workers (CHW) are expected to receive remuneration for their services. Although CMs are not classified as CHEWs or CHWs in Kenya they work in the communities. There is the need to therefore further clarify the roles and functions of CMs as health workers working in the community and to explore the opportunity presented by the Community strategy to consider incentivising CMs to reduce attrition rate.

Non remuneration of CMs by the government made CMs to charge their clients for services rendered which impedes access to skilled care for the very poor and vulnerable population. The costs of health care services including opportunity cost have been identified to impede access to skilled care. This was evident in many resource countries, in Vietnam for example, health expenditure has been estimated to have pushed about 3 million people into poverty [19, 28, 37–39]. Although CMs accept payment in kind from their clients, CMs noted that most of their clients were extremely poor and found it very difficult to pay even in kind. Instances were cited where CMs had to provide food and transport for women to return home after delivery or during transfer to health facilities.

A study showed that skilled birth attendants working in villages fear travelling at night to attend deliveries. The village midwives fearing rape refrained from visiting women at night [40]. This is also evident in this study where CMs stated that it was very difficult for them to go out at night to attend to clients. All female CMs complained of instances where people connive to rape or harm them at night. Insecurity therefore remains a big hindrance to the provision of skilled care at birth in Kenya. It is important for the Government to consider pairing CMs with Male CHEWs where such exist, as they can work together as a team with the male CHEW providing escort, health promotional activities and ensuring male involvement.

## Conclusion

An effective and efficient health system, providing quality childbirth services and access to safe EmONC are fundamental pillars for maternal and newborn health [41]. The failure to regularize formal method of remuneration for CMs as well as high cost of registration fees paid by CMs to regulatory bodies, has resulted in unregulated and arbitrary charges of clients for services provided. If the community midwifery approach is a strategy to increase access to poor and underserved women in the community, the health system should recognise CMs contribution, provide support and remuneration as the country strives to meeting Millennium Development Goals 4 and 5 by 2015.

### Study limitations

This study has several limitations. Having data only from CMs who are still within the community midwifery programme is one of the limitations of the study. CMs that left the programme could have provided insights into those factors that made them to leave. Our study also used two districts from two provinces in Kenya which could make generalizability of the findings difficult. We however sought to mitigate this by including an affluent district (Maragua) and a poor district (Kakamega). In this study, we have only presented the views of CMs and key informants, it is possible that clients might have different views. We have as part of the evaluation of the community midwifery programme, conducted focus group discussions for clients to explore their views, experiences and satisfaction with the care and services received from CMs, findings from clients will however be presented in another paper.

### Implications for policy and practice

#### Policy

To increase access to SBAs in Kenya, the following should be taken into consideration:

-  Inclusion of CMs into the Kenya Community strategy, as this will ensure that CMs are included as health workers in the community and can receive adequate remuneration for their services.
-  Introduce effective strategies to motivate and retain CMs.
-  Revision of existing policies to allow the provision of essential life-saving services by CMs such the administration of Magnesium Sulphate by CMs.

#### Practice

-  The findings of the study will provide evidence to assist the government in achieving the target of 90% of deliveries assisted by skilled birth attendants and to develop effective strategies for the improvement of maternal and newborn health [10]. It will also serve as a model for other countries with inadequate number of skilled birth attendants.
-  The Ministry of Health should create enabling environment through social insurance schemes, regulation of service charges by CMs and provision of sufficient logistics especially life-saving drugs such as Magnesium Sulphate.
-  The Ministry of Health and district health authorities should strengthen linkages between all tiers of the health service by providing communications and transport facilities.

## Electronic supplementary material

Additional file 1: **Key Informant Interview Guide.** (DOCX 17 KB)

Additional file 2: **In-depth Interview Guide.** (DOCX 19 KB)

## Abbreviations

APHIA II: AIDS Population and Health Integrated Assistance II
CM: Community midwives
EmONC: Emergency Obstetric and Newborn Care
IDI: In depth interview
KII: Key informant interview
LSS: Life saving skills
MDG: Millennium Development Goals
PSI: Population Services International
SBA: Skilled birth attendance
TBAs: Traditional Birth Attendants.
