# Supplementary material for: Opportunities and challenges in implementing community based skilled birth attendance strategy in Kenya
Source: BMC Pregnancy Childbirth. 2014 Aug 15;14:279. doi: 10.1186/1471-2393-14-279 (PMC4262243; doi:10.1186/1471-2393-14-279)
Supplement: Supplementary file 1 — Additional file 1: Key Informant Interview Guide. (DOCX 17 KB) [file 12884_2013_1244_MOESM1_ESM.docx]

**Additional file 1: Key Informant Interview Guide**

Target: officials of MoPH, RH Programme and Population Council

**Objectives:**

- Explore experiences of coordinators of the community midwifery programme
- Explore individual understanding of Skilled Birth attendance (SBAs) and maternal mortality
- Explore individual experiences with the project
- Explore factors that enhance or hinder the smooth implementation of CM Programme
- What’s the method or means of remuneration for the CMs

**Introduction:**

- Introduce the study and explain the objective of the interview
- Explain that there are no wrong answers
- Explain that if it is fine to skip a question if the participant does not feel comfortable answering it

| **Topic** | **Ideas to explore** | **Probe** |
| --- | --- | --- |
| Perception of maternal mortality | In your opinion, what is the situation of maternal and newborn health in this community | Probe district specific? Whether is an issue within the community? |
| Background information of the community programme | What were the reasons for the implementation of the community midwifery programme | Was it related to any policy (ies)? Are the reasons still relevant? What needs to be changed? What needs to be added? |
| Characteristics of community midwives | Who are community midwives? | Criteria for recruitment? |
| Experience with the community programme | How has work with the community midwives been like? | Programme in general? Good and bad experiences? Challenges? How can they be handled? |
| Maternal death | What is a community midwife required to do in case a woman dies in her care? (Antenatal period, delivery or post-partum period?) | Documentation provided e.g. notification form? Any cultural perspective to maternal death? |
| Support to the programme | Which type of support do you provide for the CMs or CM Programme? | Financial? Material? Transportation? Communication? Training? Others? |
| Service provision | In your opinion, what range of services were CMs to provide? | Is it backed by a policy? Documents? Which ones if any? |
|  | Are there any efforts made to include them in the mainstream of the public health sector? | How? In what way (s)? |
|  | Why do you think women are now opting for the services of CMs? | Whether at their homes or to the CMs houses? What is it in your opinion has changed in these CMs? |
| Determinants of relevance or acceptability of CM Model and factors that support home-based deliveries and hinder performance of CMs | Why do you think women are utilizing the services especially delivery of CMs? | Many women attend ANC services but prefer to deliver at home, why do you think is this so? |
|  | What barriers within the community that discourage women from delivery to an SBA/CM? | Distance? Cost? Attitude of health worker? Language? Drugs? Others? |
|  | In your opinion, are the women satisfied with the services provided by the community midwives? | Community perception especially men? |
| Socio-cultural factors | What economic or socio-cultural aspects in the community promote these home based deliveries? | Decision-making? Transport? Distance? Work-overload? Poverty? How? |
| Strategy to increase SBA and sustainability of CM programme | What is currently done to increase skilled birth attendance in this community? | Activities? Community participation? Involvement of men/husbands? |
|  | What in your opinion can be done to enhance the performance of the community midwives? | Inhibitive factors as well? |
|  | Who su |  |
|  | How has the health system supported the community midwifery programme? | Probe the contribution of the specific programme e.g. RH Division or NGO? |
|  | How can the CM programme be scaled-up and sustained in Kenya? | International and national support? |

Do you have any questions or comments?

Give brief summary at the end

Thank the participant for their participation. Make sure you allow the participant to discuss anything after interview has finished?
