# Supplementary material for: Opportunities and challenges in implementing community based skilled birth attendance strategy in Kenya
Source: BMC Pregnancy Childbirth. 2014 Aug 15;14:279. doi: 10.1186/1471-2393-14-279 (PMC4262243; doi:10.1186/1471-2393-14-279)
Supplement: Supplementary file 2 — Additional file 2: In-depth Interview Guide. (DOCX 19 KB) [file 12884_2013_1244_MOESM2_ESM.docx]

**Additional file 1: In-depth interview guide**

Target: community midwives living and working in the study community

**Objectives:**

- Explore experiences of community midwives
- Explore individual understanding of Skilled Birth attendance (SBAs) and maternal health and mortality
- Explore individual experiences during emergency obstetric complications and maternal death
- Explore factors that enhance or hinder their performance
- Method or means of remuneration
- Sustainability of programme

**Introduction:**

- Introduce the study and explain the objective of the interview
- Explain that there are no wrong answers
- Explain that if it is fine to skip a question if the participant does not feel comfortable answering it

***Note: When introducing this topic it is important to be very sensitive to the feelings of the participant. No participant should be forced to respond to these questions. Again reassure participants that there are no wrong answers to these questions***

| **Topic** | **Ideas to explore** | **Probe** |
| --- | --- | --- |
| Background information about community midwifery programme | Why were you recruited in the community midwifery programme? | When? By whom? Any requirement? Either educational level? Staying within the community? Why did you decide to work as a community midwife? |
| Knowledge about skilled birth attendance (SBA) | In your opinion, what do you know about Skilled Birth Attendant (SBA) | Does it relate to maternal mortality? |
| Role (s) of the community midwife | What do you do as a community midwife? | Education? Community work? Others? |
| Service provision | What are the key services you offer for women? For your clients? |  |
|  | How do women access services and care? | Cost of services? Mode of payment for services by women? If services are not paid for what will happen? |
| Supervision of CM | Do you send reports to any authority on your activity? | Is it the community? Health facility? Others? Who supervise you? How often? |
| Logistical support | What is your source of support? | In what way (s)? How often? Whom? |
| Remuneration | How does clients show appreciation for your services offered? | Is it regular? Source if any? |
| Determinants of performance | In your own opinion, what are the factors that enhance your performance? | What you need to be able to work effectively? |
|  | Have you encountered any problem (s) in your work? | Source (s) of problem? How was it addressed? |
|  | In your opinion, what do you think can stop you from working as a community midwife? | Factors that hinder your performance? |
|  | How do you think these problems can be minimised? | Prevented? |
| Job satisfaction | Would you want to continue working as a community midwife? | Satisfaction state? Why? |
| Experience | What has been your experience as a community midwife in this community? | In what way (s)? Positive? Negative? |
|  | Do you think the community value your work/services? | How? |
|  | How do you feel when you have an obstetric emergency? | Services available? Support? Transport? Communication? Link to health facility? How? |
|  | Do you have any link with health facilities or other community midwives? | Linkage? Meetings? Reports? Visits? |
| Perception of maternal death | In your opinion, how does the community feel about a woman dying in childbirth? | Socio-cultural issues? Others? |
|  | Do you have any personal experience of a maternal death? |  |
|  | How would that have been prevented? | Determinants of effective strategies? |
|  | You were working in these same health facilities, what has changed in your work that makes women to call you or come to you for maternal and newborn services? | Factors that support home-based deliveries/care in that community? Ascertain issue of staff attitude? |
|  | Why do you think these clients or women prefer to be delivered in their homes or at home or at your place? | Determinants for acceptability of services by clients? |
| Way forward and sustainability | In your opinion, would you want this project to be implemented in other regions/districts in Kenya | Why? |
|  | How do you think this project can be sustained in this community? | How? With what support? |

Do you have any questions or comments?

Give brief summary at the end

**Ending the discussion**

Try to leave the discussion in a positive way thanking the participant for their participation. Make sure you allow the participant to discuss anything after the interview has finished.
